# Kelvin Probe Force Microscopy Imaging of Plasticity in Hydrogenated Perovskite Nickelate Multilevel Neuromorphic Devices

**DOI:** 10.1021/acsnano.4c11567

**Published:** 2025-02-11

**Authors:** Tamal Dey, Xinyuan Lai, Sukriti Manna, Karan Patel, Ranjan Kumar Patel, Ravindra Singh Bisht, Yue Zhou, Shaan Shah, Eva Y. Andrei, Subramanian K. R. S. Sankaranarayanan, Duygu Kuzum, Catherine Schuman, Shriram Ramanathan

**Affiliations:** † Department of Electrical and Computer Engineering, 242612 Rutgers University, Piscataway, New Jersey 08854, United States; ‡ Department of Physics and Astronomy, Rutgers University, Piscataway, New Jersey 08854, United States; § Center for Nanoscale Materials, Argonne National Laboratory, Lemont, Illinois 60439, United States; ∥ Department of Mechanical and Industrial Engineering, University of Illinois, Chicago, Illinois 60607, United States; ⊥ Department of Electrical Engineering & Computer Science, University of Tennessee, Knoxville, 1520 Middle Dr, Knoxville, Tennessee 37996, United States; # Department of Electrical and Computer Engineering, University of California, San Diego, La Jolla, California 92093, United States

**Keywords:** Kelvin probe force microscopy, nickelates, neuromorphic computing, synaptic plasticity, memory, imaging, neural networks

## Abstract

Ion drift in nanoscale electronically inhomogeneous semiconductors is among the most important mechanisms being studied for designing neuromorphic computing hardware. However, nondestructive imaging of the ion drift in operando devices directly responsible for multiresistance states and synaptic memory represents a formidable challenge. Here, we present Kelvin probe force microscopy imaging of hydrogen-doped perovskite nickelate device channels subject to high-speed electric field pulses to directly visualize proton distribution by monitoring surface potential changes spatially, which is also supported with finite element-based electric field distribution studies. First-principles calculations provide mechanistic insights into the origin of surface potential changes as a function of hydrogen donor doping that serves as the contrast mechanism. We demonstrate 128 (7-bit) nonvolatile conductance levels in such devices relevant to in-memory computing applications. The synaptic plasticity measurements are implemented in spiking neural networks and show promising results for classification (SciKit Learn’s Iris and Wine data sets) and control (OpenAI’s CartPole-v1 and BipedalWalker-v3) simulation tasks.

## Graphical abstract

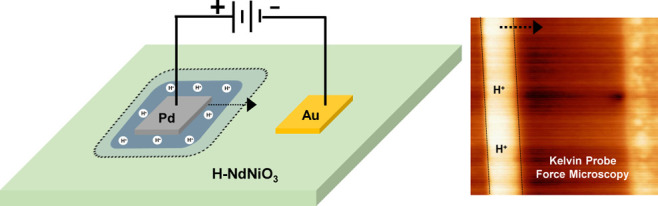

## Introduction

Ionic drift-based conductance modulation in nanoscale inhomogeneous semiconductors forms one foundational mechanism for designing neuromorphic computing hardware.^1,2^ The spatial drift of ions between electrodes either in a filamentary (i.e., localized) manner or across a significant fraction of the electrode area (i.e., nonfilamentary) results in distinct conductance levels, often in a nonvolatile manner. Therefore, the spatial profile of the ions/dopants is of fundamental importance in setting the global resistance and further controlling it by external fields. Imaging the dopant position and concentration in a nondestructive manner inside functional devices is a grand challenge and essential to advancing the current understanding of such complex semiconductors, primarily due to the lack of spatial resolution to image the dopant ion itself directly (small and light ions such as oxygen vacancies,^3^ lithium ions,^4^ protons,^5^ etc.) embedded inside a lattice, or lack of enough spatial resolution or contrast to monitor their migration after subjecting the device to fast external stimuli. The most challenging part is the ability to image the changes in the channel after brief, rapid bursts of electric field pulses that are often utilized to set various conductance levels in multilevel synaptic devices for neuromorphic computing.

Various oxide materials are being investigated as emerging semiconductors for in-memory and neuromorphic computing.^6^ Among these candidates, perovskite rare-earth nickelates^7^ (RNiO_3_, where R represents a rare-earth element, e.g., Nd, Sm) have garnered significant interest^8^ as hardware for neuromorphic computing owing to the large tunability in electrical resistance arising from hydrogen donor doping.^9–11^ The electrical resistance of perovskite nickelates, such as NdNiO_3_ (NNO), can be increased by many orders of magnitude by doping with hydrogen donors^12^ owing to a half-filled *e*_*g*_^2^ configuration of the Ni d-orbitals^13^ due to electron injection. This phenomenon has opened a new paradigm for exploring novel electronic phases and applications in emerging field-controlled electronic devices.^14^ Interstitial sites are occupied by protons that migrate under the influence of electric fields. Hydrogenation of the catalytic Pd electrode (Pd–NNO–H_2_) at the triple-phase boundary causes the incorporation of protons into the NNO crystal lattice.^15^ Controlling the local concentration and spatial distribution of protons by electric fields enables tuning the resistance states. Therefore, it is crucial to understand the microscopic drift-diffusion phenomena concerning protons in the lattice, as they govern various neuromorphic properties. Although there have been studies on measuring global channel resistance dependence on electrical stimulus in protonic devices,^16^ nondestructive imaging of the channel region in a working device, especially in a spatially resolved manner, is still in its infancy. Changes in charge concentration gradients happen over microscopic length scales,^17^ where even slight variations can impact the output signal of the instruments. Thus, experimental techniques with high resolution and sensitivity are sought to probe the localized charge and electronic structures. Previous works along these lines include infrared nanoimaging of the dielectric environment^18^ (contrast mechanism sensitive to free carrier density), electron energy loss spectroscopy in a scanning transmission electron microscope (contrast mechanism sensitive to unoccupied density of states),^19^ and synchrotron fluorescence imaging (contrast mechanism sensitive to charge transfer-based spectral weight). Secondary ion mass spectroscopy and nuclear reaction analysis have also been utilized to monitor hydrogen doping in the literature.^20,21^ However, it is challenging to use such techniques to monitor active device channels in a spatially resolved manner. Kelvin probe force microscopy (KPFM), a scanning probe microscopy technique, provides a powerful lab-scale capability to investigate electronic phenomena at the nanoscale using a distinct contrast mechanism sensitive to work function.^22–24^ KPFM measures the Coulomb force between the surface and the conductive atomic force microscope (AFM) tip to access the electrical properties of the samples, such as work function in terms of contact potential difference (*V*_CPD_).^25,26^ This technique has been utilized to measure the work function for metal samples quantitatively,^27^ surface potential for dielectrics,^28^ and band bending in semiconductors.^29^ KPFM has also been used to study dopant profiles in semiconductors,^30,31^ photogenerated carriers in optical devices,^32^ switching characteristics attributed to field-induced agglomeration of ionic vacancies,^33^ nanoscale conductive channel formation,^34^ surface photovoltage,^35^ or dynamic modulation of the Schottky barrier.^36^ In the current work, we have quantitatively probed the change of work function in protonated NNO films using KPFM and successfully measured the drift distance of protons under electric field pulsing. The NNO film was synthesized on a lanthanum aluminate (LAO) single crystal substrate by sputtering. Asymmetric electrodes with gold (Au) and palladium (Pd) were patterned on the thin film by using photolithography techniques. Subsequent heating of the thin film in argon/hydrogen forming gas causes about a 10^3^-fold increase in the electrical resistance of the NNO film, now referred to as hydrogenated NNO or HNNO. Pd acts as a catalyst for H_2_ adsorption and dissociation. Thus, the density of protons is much higher near the Pd electrodes and they drift under an electric field stimulus. From KPFM experiments coupled with pulsed electrical measurements, simulation of the electric field distribution with COMSOL, and first-principles calculations, we find that the migrated proton clouds result in distinct resistance states, which can be controlled by voltage amplitude, pulse width, and number of applied electrical pulses. The proton cloud drift directly affects the material’s work function, supported by first-principles calculations. The change in the work function results in a change of the contact potential difference between the sample surface and the KPFM tip, providing a contrast mechanism for imaging. The results open new directions for investigating nanoscale doping profiles of protonic neuromorphic devices using the KPFM technique. The tunable proton drift enables the demonstration of 128 nonvolatile conductance levels. These states are created by the migration of proton clouds with applied electrical pulses. As a proof-of-principle application, the synaptic plasticity measurements have been implemented in spiking neural networks and show promising results for classification (SciKit Learn’s Iris and Wine data sets) and control (OpenAI’s CartPole-v1 and BipedalWalker-v3) simulation tasks.

## Results and Discussion

X-ray diffraction (XRD) data of the LAO substrate, NNO, and HNNO are shown in the Figure S1 of the Supporting Information. The LAO (002) XRD peak is visible in all samples, while the characteristic NNO peak (shown in blue) shifts to a lower 2θ after hydrogenation (shown in red). Out-of-plane lattice constant values for NNO and HNNO are calculated to be 3.826 and 3.832 Å, respectively. A microscope image of the device under test (DUT) is shown in Figure 1a. The square at the center is a Au electrode, surrounded by four Pd electrodes. The channel electrical resistance is sensitive to the concentration and distribution of doped protons within the lattice. Atomic force microscopy images of the device at 3 different channels, i.e., between the Au electrode and Pd1, Pd2, and Pd3, are shown in Figure 1b, with visible *z*-scale and *xy*-scale. After being subject to hydrogenation, the device exhibits an almost 10^3^ times increase in electrical resistance, as shown in Figure 1c. Note that control experiments performed with Ni electrodes did not show any appreciable change in the device resistance after similar hydrogenation experiments. This indicates the role of Pd in dissociating and incorporating hydrogen into the nickelate lattice. The potentiation, depression, and learning rates of an individual synaptic junction can be systematically controlled by pulsed voltage bias.^37^ Figure 1d shows the potentiation-depression cycle at different pulse voltages (positive bias and negative bias denote potentiation and depression, respectively) and widths. It is observed that the resistance of the device increases when a positive voltage pulse train is applied to the Pd electrodes, with respect to the Au electrodes.

**Figure 1:**
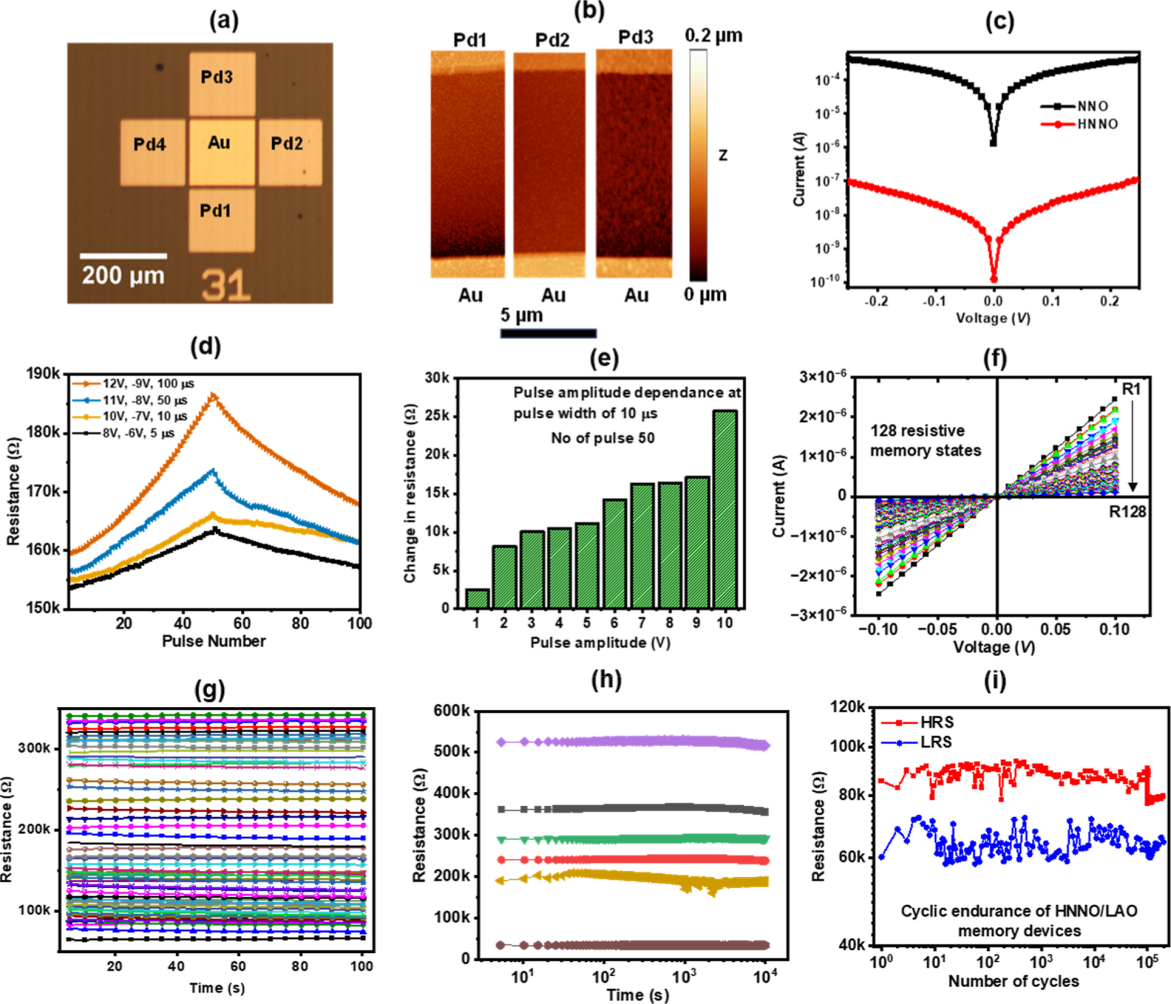
Electrical characteristics of a representative device. (a) An optical microscopy image of the DUT. At the center, there is an Au electrode surrounded by four Pd electrodes at a gap of 10 μm. (b) AFM micrograph of the channel between Au and Pd1, Pd2, and Pd3 electrodes, respectively, with both *z* and *xy* scales visible. (c) *I*–*V* characteristics showing an ∼10^3^ times increase in resistance after hydrogenation. (d) Potentiation-depression cycle at different pulse voltages and pulse widths. (e) Dependence of the potentiation with increasing voltage pulse height. (f) *I*–*V* data of 128 distinct resistance states in HNNO via different parameters of electrical stimuli. (g) Retention of these multilevel resistive memory states over a time period of 10^2^ seconds. (h) Long-term retention study of six resistance states over 10^4^ seconds. (i) Endurance of the devices over 2 × 10^5^ number of cycles of switching between two memory states measured at set voltage 10 V, reset voltage −10 V, pulse width 10 μs, and a read voltage of 0.1 V.

Similarly, resistance decreases when a negative voltage pulse train is applied at Pd. This sensitivity allows a wide range of resistance states to be achieved by applying different combinations of pulse heights and widths. The electric-field-driven resistance modulation can be attributed to the drift of interstitial protons in the HNNO lattice, which suggests that the application of a positive bias causes the expansion of the HNNO region due to proton migration, and applying a negative bias causes the proton-rich region to shrink.

These potentiation-depression data in Figure 1d have been fitted with Box-Lucas formulas (Figure S2)^37^ for the nonlinearity factor (β).

1 $$\text{For}\;potentiation:R_{pot}=\alpha \left(1-e^{-\beta n}\right)+R_{\min}$$

2 $$\text{for}\;depression:R_{dep}=\alpha \left(1-e^{\beta \left(n-n_{\max}\right)}\right)+R_{\max}$$

where *R*_pot_ and *R*_dep_ are the resistance data in potentiation and depression cycles, respectively, and *n* is the normalized pulse number. The constants α and β depend on the normalized pulse numbers *n*_max_ and the maximum and minimum resistances (*R*_max_ and *R*_min_) for each potentiation-depression cycle.

3 $$\alpha =\frac{R_{\max}-R_{\min}}{\left(1-\exp^{\left(-\beta n_{\max}\right)}\right)}$$

The fitted data for all of these potentiation-depression cycles have been provided in Figure S2 of the Supporting Information. It may be noted as the value of β decreases, the plot becomes more linear.

In Figure 1e, we have shown different measurements where the change in resistance gradually increases when the height of the electrical pulse is increased. Thus, a combination of experimental parameters concerning voltage amplitude, cycle number, etc. can be chosen to demonstrate the efficacy of synaptic plasticity in network performance. As proof of concept, 128 nonvolatile conductance levels are shown in Figure 1f within a useful range of resistance (roughly 60 to 600 kΩ). These nonvolatile levels are distinct, and retention of resistance is presented for 10^2^ seconds, as shown in Figure 1g. Figure 1h exhibits the retentivity of six representative states for an extended period of 10^4^ seconds. The cyclic endurance of a representative device over 2 × 10^5^ cycles is shown in Figure 1i.

We utilized KPFM to understand the distribution of proton clouds after the device is subject to electric pulses. To study the change in the spatial map of *V*_CPD_ upon electrical pulsing, a series of 20 electric pulses with an amplitude of 10 V (and −7 V) and a width of 10 μs were applied to the Au–Pd1 junction. Using 20 pulses under our experimental conditions was necessary because the proton migration resulting from a single short pulse is too small to be detected using scanning techniques in a working device. The resulting spatial map of *V*_CPD_ for the HNNO film is shown in Figure 2a–f for the Au–Pd1 junction, Figure 3a–f for the Au–Pd2 junction, and Figure S4a–f of the Supporting Information for the Au–Pd3 junction, respectively. The data depict the spatial map of measured *V*_CPD_ for both NNO and HNNO films. Before any electrical pulsing, denoted as the “initial state”, the spatial extent of hydrogen doping in the HNNO film is observed to diminish away from the Pd electrode triple phase boundary due to the diffusive nature of doping. A distinct difference in the *V*_CPD_ profile near the Pd electrode for the HNNO film is attributed to hydrogen doping, with the bright contrast in KPFM mapping visualized in 3D near the Pd electrode indicating the areal extent of the hydrogen doping, which eventually decays over a specific length scale.^38^

**Figure 2:**
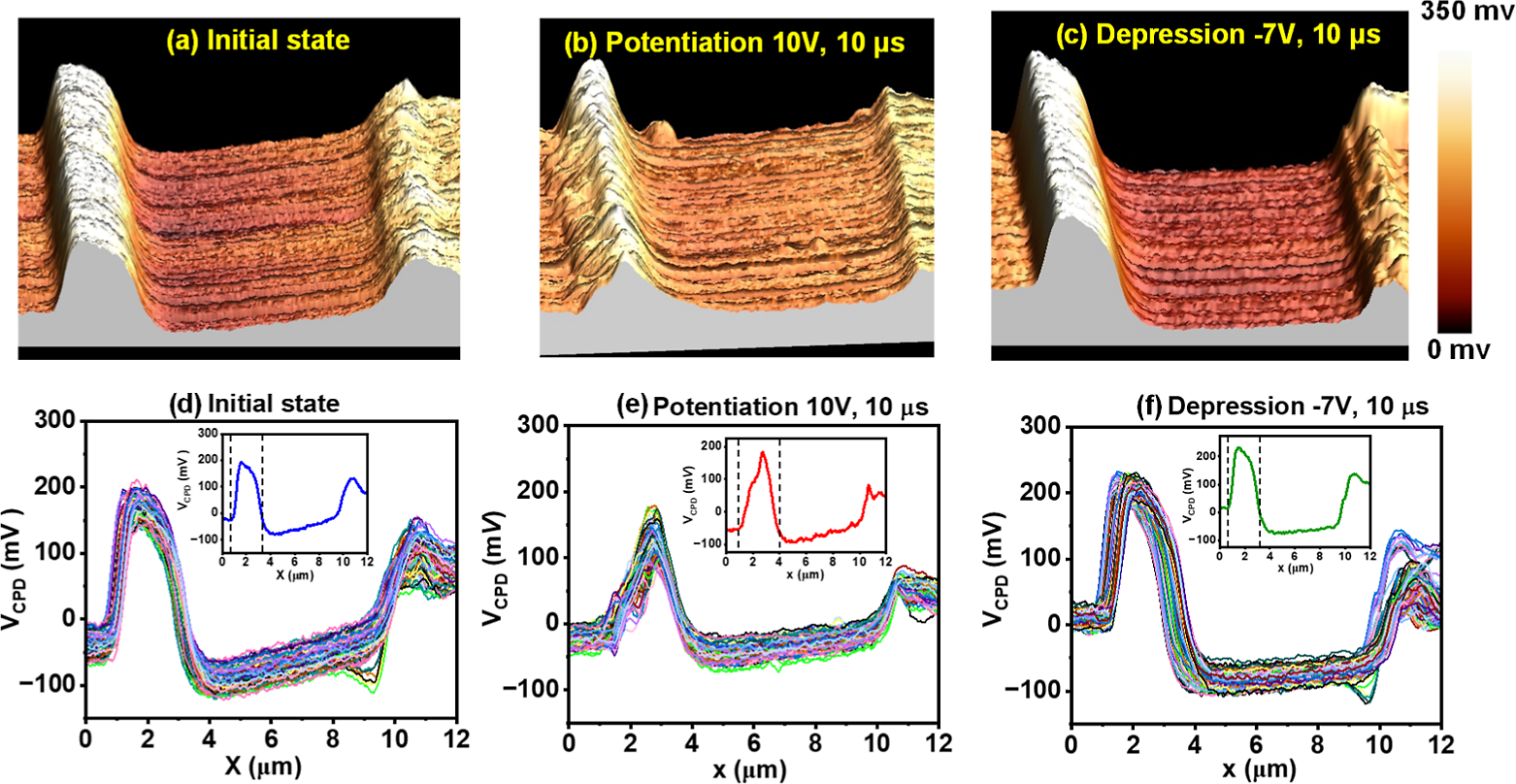
KPFM scan at the Au–Pd1, i.e., the same junction where electrical programming is done. The left side and right side of the KPFM maps correspond to regions surrounding the Pd1 and Au electrodes, respectively. Mapping of contact potential difference (*V*_CPD_) between the KPFM tip and the sample visualized in 3D for (a) the pristine sample before any electrical programming, termed as “initial state”, (b) after applying 20 pulses of 10 V height and 10 μs width, termed as “potentiation”, (c) after applying 20 pulses of −7 V height and 10 μs, termed as “depression”. (d–f) Hundred line profiles showing variation of *V*_CPD_ within the channel between Au and Pd electrodes at distinct positions for initial, potentiation, and depression conditions each. Insets of (d–f) show one representative line profile for each case, where the dotted lines indicate the edges of heavily H-doped regions.

**Figure 3:**
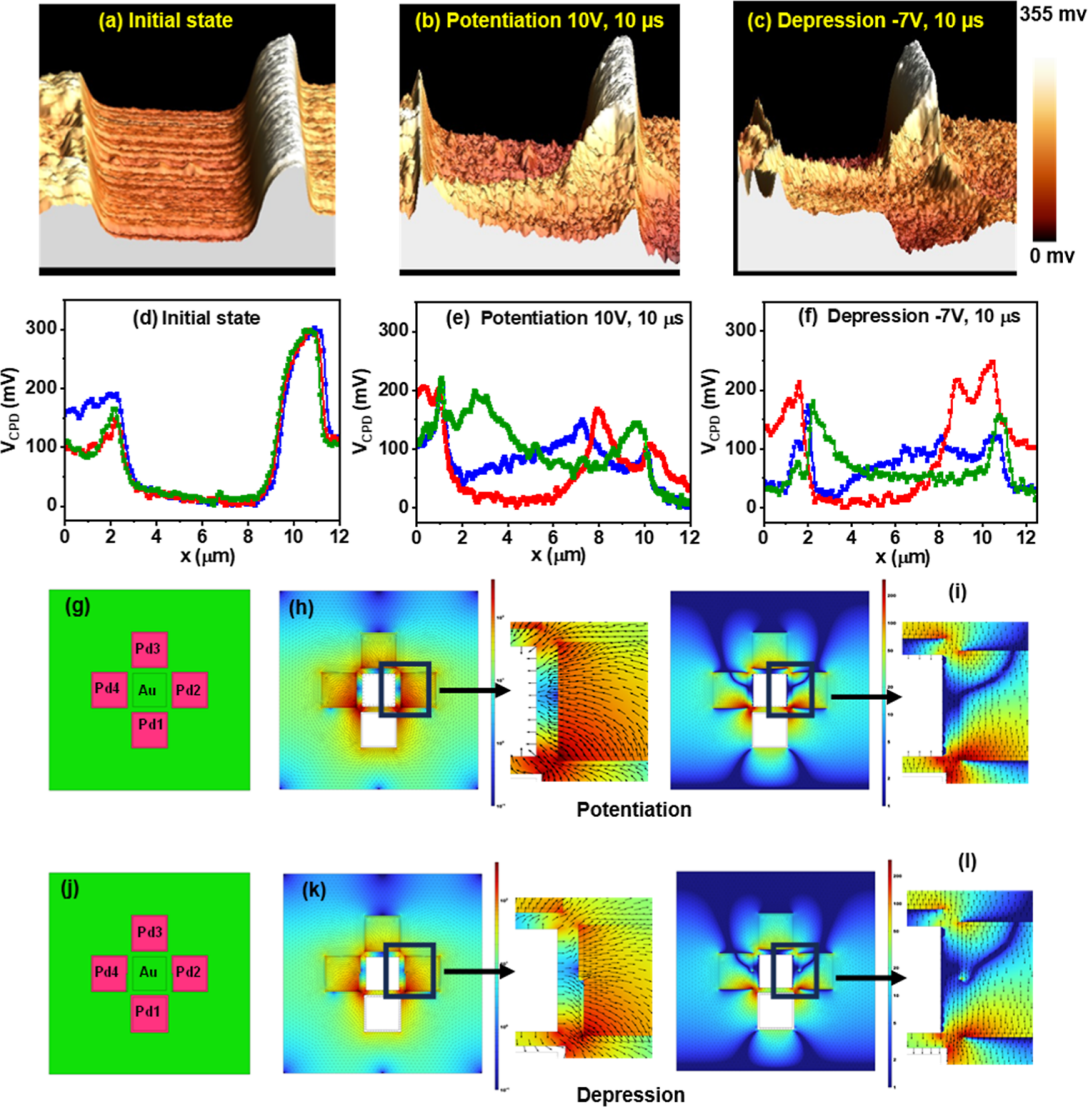
KPFM scans at the Au–Pd2 junction, while the electrical programming is only done at Au–Pd1. The left side and right side of the KPFM maps correspond to regions surrounding the Au and Pd2 electrodes, respectively. Mapping of contact potential difference (*V*_CPD_) between the KPFM tip and the sample visualized in 3D for (a) the pristine sample before any electrical programming, termed as the “initial state”, (b) after applying 20 pulses of 10 V height and 10 μs width, termed as “potentiation”, (c) after applying 20 pulses of −7 V height and 10 μs, termed as “depression”. (d–f) 3 numbers of line profiles showing variation of *V*_CPD_ within the channel between Au and Pd electrodes at 3 distinct positions for initial, potentiation, and depression conditions each, respectively. COMSOL simulations of the device in potentiation and depression cases. (g) Proton cloud distribution for the potentiation case in a pristine device. (h) Electric field distribution during potentiation. (i) Distribution of the Y-component of the electric field in the potentiation case. (j) Proton distribution for the depression case in a potentiated device as observed in KFPM scans. (k) Electric field distribution during depression. (l) Distribution of the Y-component of the electric field in the depression case.

Figure 2a–c presents KPFM mapping of the samples at the initial condition, after applying 20 pulses of 10 V height and 10 μs width, termed as “potentiation”, and after applying 20 pulses of −7 V height and 10 μs, termed as “depression”. The left side is the Pd electrode; therefore, naturally, the proton cloud is concentrated in this region compared to the Au electrode on the right side. The vertical scale shows color mapping. Compared to the initial state, the proton cloud near the Pd electrode loses height (from the change in color) and spreads out after applying positive bias electric pulses. Again, applying negative bias electric pulses causes the proton cloud to shrink back and regain its height. Thus, we infer that the electrical stimuli can influence the proton distribution in a controlled and reversible way. Figure 2d–f shows 100 different line profiles, showing a variation of *V*_CPD_ within the channel between Au and Pd electrodes at distinct positions for initial, potentiation, and depression conditions, respectively. The line profiles are somewhat similar barring some local fluctuations, suggesting reliable estimation of proton cloud edges. Insets of Figure 2d–f show one representative line profile for each case, where the dotted lines indicate the edges of heavily H-doped regions. The width of the proton clouds was calculated by averaging 100 line profiles for each case.

From the statistical analysis of 100 line profiles around the Pd electrode, we estimated the width of the heavily proton-doped region to be ∼2.7, 3.0, and 2.6 μm for the initial state, potentiated, and depressed states, respectively. The results suggest that the proton cloud expands under positive electrical pulses and shrinks under negative electrical pulses. The synaptic electrical characteristics of the device under similar potentiation-depression measurement conditions have previously shown reversible trends in Figure 1d. Therefore, a clear correlation between the drift of proton clouds, which causes local changes in proton concentration, and the resistance states, is observed.

To better understand the correlation between proton drift and *V*_CPD_, we performed a KPFM scan at the adjacent junction Au–Pd2. Note that there was no electrical stimulus applied directly to this junction. Also, this junction is further away; thus, the proton cloud profile should result in different KPFM profiles. KPFM visualization and line profiles of the Au–Pd2 junction are shown in Figure 3a–f. The data show interesting phenomena happening locally: the proton cloud near the Pd2 electrode is observed to drift away in an inhomogeneous fashion when electrical stimuli are applied at the Au–Pd1 junction.

We performed COMSOL simulations for the device structure in both the potentiation and depression cases to understand the spatial distribution of the electric field in the nickelate film. The results are shown in Figure 3g–l. We studied a pristine device in the potentiation case, assuming a uniform proton cloud thickness of 2 μm around all of the Pd electrodes initially. A positive bias was applied to the Pd1 electrode, while the Au electrode was grounded, as shown in Figure 3g. Figure 3h illustrates the electric field distribution for the device in the potentiation case. Figure 3i shows the distribution of the Y-component of the electric field in the same case. The lower halves of the Au–Pd2 and Au–Pd4 junctions have an upward-pointing electric field of higher magnitude.

In contrast, the upper halves of these junctions have a downward-pointing electric field of lower magnitude, leading to the proton cloud being pushed upward. This nonuniform nature of electric field distribution in the Au–Pd2 junction, with higher intensity near the lower parts, causes the abrupt disruption of the proton cloud around the Pd2 electrode, as seen from the KPFM data in Figure 3b. The Au–Pd1 and Au–Pd3 junctions exhibit upward and downward pointing electric fields, causing the proton cloud to move upward and downward accordingly. The change in proton cloud distribution at the Au–Pd3 junction is lower due to a weaker electric field, similar to what we have observed in the KPFM data of the Au–Pd3 junction in Figure S4, which will be discussed in the following section.

In the depression case, we examined a potentiated device using the proton cloud distribution observed from the KPFM scans, as shown in Figure 3j–l. Pd2 and Pd4 were assumed to have the proton cloud present only in the upper half of the electrode at the Au junction. A negative bias was then applied to the Pd1 electrode, as depicted in Figure 3j. Figure 3k illustrates the electric field distribution for the device after negative pulses of −7 V and 10 μs width are applied. Figure 3l shows the distribution of the Y-component of the electric field in the same case. The lower halves of the Au–Pd2 and Au–Pd4 junctions have a downward-pointing electric field of a higher magnitude.

Conversely, the upper halves of these junctions have an upward-pointing electric field of a lower magnitude. Since most of the proton cloud is distributed in the upper half of the junctions (observed in the KPFM scan), which is experiencing a weak upward-pointing electric field, there is very little change in the proton cloud distributions at the Au–Pd2 junction, which explains the absence of visual changes of the proton cloud in KPFM Figure 3c when a negative pulse train is applied. The Au–Pd1 and Au–Pd3 junctions exhibit downward- and upward-pointing electric fields, causing the proton cloud to move downward and upward accordingly. The change in proton cloud distribution at the Au–Pd3 junction is lower due to a weaker electric field. A strong correlation between *V*_CPD_ values observed from KPFM at the Au–Pd2 junction and the electric field is observed, as shown in Figure S3a–h of the Supporting Information, along with related discussions. The correlation is higher near the vicinity of the Pd electrode, which implies the applied electric field resulted in proton displacement in the heavily doped regions.

KPFM mapping and line profiles taken at the further located Au–Pd3 junction are shown in Figure S4a–f of the Supporting Information. Electrical stimulus at Au–Pd1 does not affect KPFM signals nearby Au–Pd3, contrary to what has been observed at Au–Pd1 and Au–Pd2, due to the spatial separation between Au–Pd1 and Au–Pd3 junctions (more than 100 μm). When a positive pulse train is applied at Au–Pd1, the proton clouds drift toward the Pd3 electrode due to electrostatic repulsion from the positive bias at the Au–Pd1 junction, which is reversible when a negative pulse train is applied at Au–Pd1. From Figure S4d–f, the width of the proton cloud remains unaffected mainly, although minor changes in the values of *V*_CPD_ are observed. After positive pulsing at Au–Pd1, *V*_CPD_ is slightly diminished at the Au electrode since protons are “pushed away” toward the Au–Pd4 junction by electrostatic repulsion. Yet, the value of *V*_CPD_ is almost unaffected at the Pd4 electrode, indicating the diminishing effect of the electric field on proton concentration with distance.

From these results, we see a correlation between the increased resistance of the device and the greater proton distribution, which increases the contact potential difference (*V*_CPD_) between the KPFM tip and the surface, thus allowing us to analyze and map the dopant profile. Contact potential (*V*_CPD_) is related to the work function of the sample (Φ_sample_) according to the following relation.

4 $$V_{CPD}=\frac{\Phi_{probe}-\Phi_{sample}}{e}$$

The terms e, *V*_CPD_, Φ_probe_, and Φ_sample_ represent the electronic charge, contact potential difference, the work function of the tip, and the work function of the sample respectively, implying that with increasing dopant concentration, *V*_CPD_ increases, which in turn decreases the work function of the sample, making it more negative.

From the KPFM and COMSOL data, we can infer that positively charged protons are driven in the direction of the electric field, thus changing the local concentration of H atom per unit cell, which will, in turn, locally change the work function and, therefore, the surface potential of the sample, which the KPFM is able to sense quantitatively. First-principles calculations of the changes to the work function of NNO doped with various concentrations of hydrogen were carried out to understand the nontrivial experimental trend of surface potential changes with doping concentration. The work function was evaluated for NNO surfaces oriented along the (100) direction, with the surface termination chosen to be symmetric and nonpolar (SI Figure S5). Since the absolute value of the work function depends on the choice of surface termination, here, we focus on the relative changes to the work function due to hydrogen dopant concentration, which allows us to understand the experimental trends from KPFM imaging. The work function change (ΔΦ) induced by H doping displays a corresponding monotonic decrease from −1.2 eV with increasing H doping from 0 to 0.5H per Ni atoms, as shown in Figure 4a. The reduction in the work function with increasing H doping can be attributed to an increase in electron concentration resulting from charge transfer from hydrogen to the NNO surface layer.

**Figure 4:**
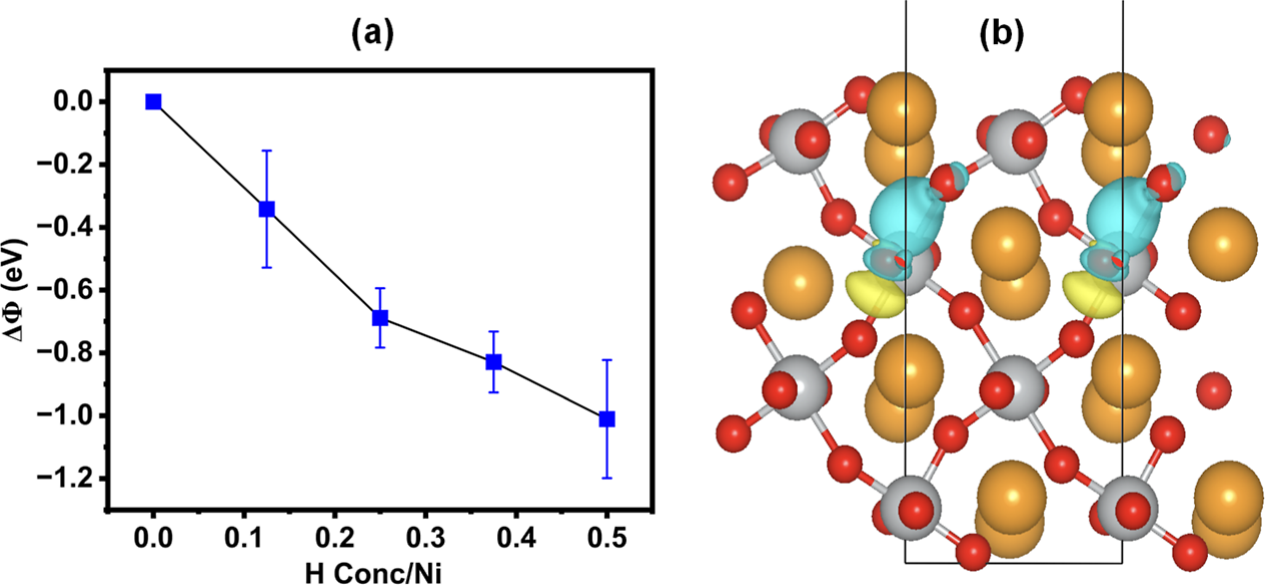
Effect of hydrogen doping on work function from first-principles. (a) Change in work function (ΔΦ) of NNO with increasing hydrogen concentration (per Ni atom). Density functional theory (DFT) calculations for NNO (100) surfaces reveal a consistent decrease in ΔΦ, reaching −1.2 eV as H doping increases from 0 to 0.5H per Ni. This trend indicates work function reduction due to electron donation from hydrogen, elevating overall electron concentration. (b) Charge density redistribution upon single hydrogen addition to NNO. Yellow regions indicate increased electron density, while cyan shows decreased density. Enhanced electron presence near the hydrogen site and diminished density between Ni and O atoms corroborate hydrogen’s electron contribution. This redistribution augments electron concentration and consequently lowers the work function.

Charge density difference plots visualizing the changes in electron density before and after H inclusion in NNO are shown in Figure 4b. Yellow indicates electron accumulation in these plots, and cyan represents electron depletion. The accumulation of electron density around the H site and depletion of electron density between the Ni and O atoms confirm electron donation from H, increasing the electron concentration and reducing the work function. The almost linear relationship between ΔΦ and H concentration suggests that the work function can be modulated by controlling the amount of incorporated hydrogen, which can be better understood from Figures S6 and S7 of the Supporting Information. The effect of H doping on the ionic character of the O bonds in NNO was analyzed. As shown in Figure S8 of the Supporting Information, the bond ionicity of the O atoms decreases monotonically as the H dopant concentration increases from 0 to 0.5 per Ni atom. The bond ionicity is defined as the ratio of the Bader charge on O to the −2 formal charge of O in a purely ionic system. The increasingly positive Bader charges indicate that the O–Ni bonds become more covalent and less ionic with more significant H doping. This reduction in ionic character is attributed to electron donation from the H dopants, increasing the O–Ni bond’s covalency. KPFM experiments show a decrease in work function with H addition, consistent with the decreasing trend predicted by our DFT calculations.

As a proof-of-principle use case of the tunable resistance levels arising from proton distribution, we examined the plasticity in network-level tasks. In order to capture the device behavior, deep-learning models for each device type were created to determine updated synaptic plasticity values based on previous readings and applied voltage. The models allow for the integration of the device characteristics to be applied to neuromorphic applications, such as classification and control tasks. The classification results for the Iris and Wine data sets are presented in Figure 5a–d. It can be seen that the testing accuracies for all of the devices are relatively similar. The device results are compared against networks without spike time-dependent plasticity (STDP) to act as a control for the experiment. These non-STDP networks also performed similarly to the device models, which is to be expected since STDP tends to perform better in noisy situations and significant variations in initial states (which is not portrayed by the classification tasks) since the STDP rules can enforce behaviors based on the repetition of neuron fires without Evolutionary Optimization for Neuromorphic Systems (EONS) having to fine-tune for it. Still, the 15 μm gap device resulted in a network performing best for Iris and Wine data sets, including networks without STDP, which suggests the plasticity data is better suited for these applications.

**Figure 5:**
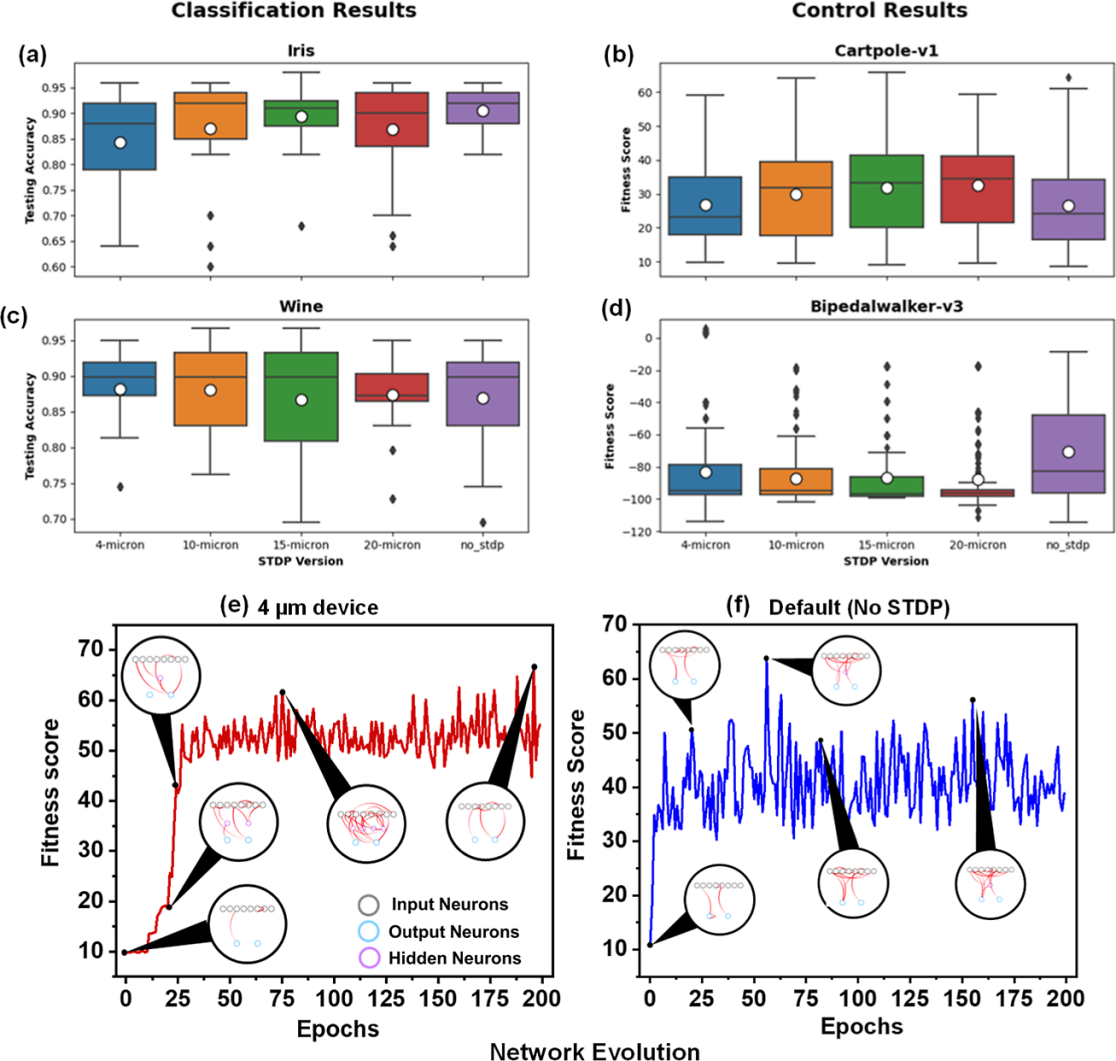
Application results for each device type. Classification results using (a) Iris and (c) Wine data sets, and control results using (b) Cartpole-v1 and (d) Bipedalwalker-v3. Network evolution comparing (e) 4 μm device and (f) default (no STDP) configuration for CartPole-v1 application.

The control task results were found to be more variable. The CartPole-v1 results demonstrate how the average performance increases as the distance between the gold–palladium nodes increases, with the 20 μm device performing the best and showing noticeable improvement over networks without STDP, clearly demonstrating a trade-off between performance and junction gap. On the other hand, the BipedalWalker-v3 application, on average, shows no improvement in the presence of STDP. However, the 4 μm device did have an outlier that produced the best-performing network for the application. Again, this disparity can be attributed to the initial conditions of the control tasks. The CartPole-v1 application has numerous variations in the initial states (the position and velocity of the cart and pole) against which STDP can be leveraged; however, BipedalWalker-v3 has a more consistent initial setup and is a more complex application. Figure 5e,f demonstrates an example of network evolution for a single run of the CartPole-v1 application, comparing the 4 μm device to the default configuration without STDP.

From Figure 5e,f, we can see that the fitness scores of the networks improve over time in subsequent epochs. Most noticeably, we can see that the 4 μm device, on average, yields better fitness scores compared to the default, thus suggesting that the STDP capabilities of the 4 μm device are more fitting for this particular application. The smaller channel length may have resulted in a better modulation of synaptic weight in this case. This result is consistent with the expectation that STDP is typically better suited for noisy and temporal situations, such as control tasks.

Additionally, we showcase the network structure at key points in the evolution process to highlight how EONS modifies the networks over time. Through EONS, the population of networks is evolved by adding additional synaptic connections, hidden neurons, or modifying various parameters (delay, thresholds, etc.), which is clearly seen in Figure 5e,f, where initially the networks are simple with few synaptic connections, which evolve more complex structures over time as the algorithm converges to a solution. Interestingly, the 4 μm device eventually reverts to a simple network with no hidden neurons and fewer synaptic connections compared to the default configuration without STDP. A possible explanation for this can be that the 4 μm device’s STDP behaviors are able to better adapt to the complexity of the environment without needing a more complex network structure to process the inputs. The lack of STDP rules for the default configuration is possibly compensated through its more complex network structure. This explains the disparity in network structures of the later epochs of the 4 μm and default configurations. This property can be further examined in the future in creating smaller networks for neuromorphic hardware, which can potentially help in routability and potential energy efficiency due to fewer neurons and synapses.

## Conclusions

Kelvin probe force imaging is demonstrated as a suitable technique for studying proton-drift-based synaptic plasticity in operando perovskite nickelate devices subjected to fast electric field pulses. Trends in surface potential changes are consistent with first-principles calculations of work function variation with doping. We demonstrated 128 nonvolatile conductance levels by modulating proton concentration with electric fields. Implementing the plasticity characteristics into artificial neural networks demonstrates proof-of-principle use cases for classification and control tasks.

## Methods

### Synthesis of NNO Films and Device Fabrication

An NNO film was deposited on LaAlO_3_ (LAO) substrates following the process as reported in the literature.^37^ Briefly, radio frequency (RF) magnetron sputtering was employed to deposit 50 nm thick NNO thin films on LAO (100) single crystal substrates at ambient temperature. The RF and direct current (DC) power settings were calibrated to 140 and 79 W, respectively, with argon and oxygen gas flows controlled at 40 standard cubic centimeters per minute (sccm) and 10 sccm, respectively. The substrate holder rotated at 25 rpm to ensure uniform film deposition. Postdeposition, the films underwent annealing at 500 °C for 24 h in air. Asymmetric NNO devices were fabricated by using photolithography. Gold (Au) and palladium (Pd) were deposited by using e-beam sputtering. Then, the samples were put inside a tube furnace and annealed at 115 °C for 20 min in a hydrogen/argon (10%/90%) atmosphere with a flow rate of 35 sccm, yielding HNNO thin films. The inert and noncatalytic nature of the Au electrode ensured that hydrogen doping was localized in proximity to the Pd electrodes, where hydrogen dissociation takes place.

### Device Characterization

We used a Keithley 4200A-SCS semiconductor parameter analyzer equipped with a probe station for the electrical characterization of the devices. Potentiation-depression, endurance, retention, and multistate conductance level measurements were performed by applying electrical pulses using a remote preamplifier, 4225-RPM and pulse unit 4225-PMU.

### Kelvin Probe Force Microscopy

An NT-MDT Solver Next AFM with a monocrystalline silicon HA_NC tip with a radius of curvature less than 10 nm and a spring constant of 12 N/m (K-Tek Nano HA_NC_Au) was utilized for the measurements. The sample is kept electrically grounded, and the tip is operated at an AC modulation of 0.5 V at a frequency of 114 kHz. A standard dual-pass technique was used for KPFM experiments.^39^ In this method, the first step involves measuring the surface topography using the tapping mode. During the second step, the contact potential difference (*V*_CPD_) is calculated after the tip is retracted by Δ*z* = 10 nm from the surface.

### COMSOL Simulation

The spatial distribution of the electric field was calculated for the devices under potentiation and depression cases by using the COMSOL AC/DC solver module. Solving the 2-dimensional drift-diffusion equations for a nickelate film determined the electric field distribution, assuming a configuration similar to that of the experimental device under study.

### First-Principles Calculations

The Vienna ab initio Simulation Package (VASP) was utilized to perform first-principles DFT calculations to investigate the work function of pristine and H-doped NNO (100) surfaces. The generalized gradient approximation of Perdew–Burke–Ernzerhof was used as the exchange–correlation functional.^40^ To properly account for the strong on-site Coulomb interactions of the localized Ni 3d electrons, DFT + *U* corrections were included with *U* = 4.6 eV and *J* = 0.6 eV for Ni atoms.^18^ The surfaces were modeled as periodic slabs oriented in the (100) direction to match the experimental samples. The slabs had a thickness of 10 Å in the *z*-direction and were separated by 15 Å of vacuum to avoid spurious interactions between periodic images. The surface structures were initially optimized by relaxing the atomic positions using the conjugate gradient method^18^ until the residual forces on all atoms were less than 0.01 eV/Å. The plane wave cutoff energy was set to 540 eV, and the electronic energy convergence threshold was 10^–5^ eV for the relaxations. Spin polarization was included with calculations performed at the Γ-point using a *k*-point mesh of 1000 *k*-points per reciprocal atom. In addition, symmetry was turned off, and AMIX = 0.1 and BMIX = 0.001 mixing parameters were used to improve the electronic convergence. After the relaxed geometries were obtained, the work function was calculated for the pristine and H-doped NNO (100) slabs. H atoms were doped at interstitial sites near O atoms for varying concentrations.

The work function is the minimum energy required to remove an electron from the surface of a solid material to a point just outside it in a vacuum. This energy can be mathematically described by the equation *W* = *V*_vacuum_ – *E*_F_. *V*_vacuum_ represents the vacuum level, which is the electrostatic potential energy of an electron at rest in the vacuum near the material’s surface. *E*_F_ denotes the Fermi energy of the material, which is the highest energy level occupied by electrons at the absolute zero temperature. Figure S6 illustrates this energy barrier, demonstrating that *V*_vacuum_ is attained when the electron is sufficiently far from the surface, ensuring that the potential remains stable over a small range in the vacuum. This methodology for determining the work function has been widely utilized in prior research.^41–44^

Work functions are calculated using NNO (100) slabs that have varying concentrations of H atoms, including pristine NNO, 0.125H per Ni, and 0.25 per Ni, as shown in Figure S7 of the Supporting Information.

### Spike Time-Dependent Plasticity

The proposed gold–palladium devices depict strong STDP behavior, making them a promising candidate for neuromorphic systems. To benchmark the impact of these devices as they relate to STDP for SNNs, an exploration of 4 variations of the device was performed. The distance between the gold and palladium nodes is varied between 4, 10, 15, and 20 μm to explore how the resulting potentiation-depression curves impact the performance of SNNs on various applications. Physical devices at the varied ranges were fabricated, and experimental resistance readings were recorded based on an applied potentiation voltage of 10 V, depression voltage of −7 V, and a pulse width of 10 μs. The potentiation and depression states were swapped every 20 samples (by applying the appropriate potentiation or depression voltage) for a total of 160. Individual device models were created to capture the STDP behavior by utilizing the resistance readings from the four devices. The models can predict the next resistance value of the respective device based on the applied pulse width, voltage, and previous resistance, thus allowing us to model the resistance for an arbitrary number of samples with varying periods of potentiation and depression, making them robust when integrated into SNNs to model synapse behaviors. These models were created by using deep neural networks (DNNs) from traditional deep learning. DNNs were chosen to model the devices since they excel at regression tasks and can approximate any arbitrary function regardless of complexity through the Universal Approximation Theorem.

For this work, the DNN consists of an input layer followed by seven dense feedforward layers of size 512, 256, 128, 64, 32, and 16, 1. Each layer utilized the ReLU activation function and was trained for 400 epochs with RMSE as the loss function. An example of a device model’s STDP curve compared against experimental readings is shown in Figure S9a–d for different channel widths. Note that the model takes in the resistance value of a previous sample to predict the following sample; therefore, slight deviations can grow larger in subsequent samples. However, the model captures the general trends of the curves, which is more important for the behavior of the device within an SNN. This behavior is further reinforced since the resistance value occurring in the kilo-Ohm range will be mapped to a value between −10 and 10 for the synaptic weight within the SNN. This reduction in resolution will reduce the impact of the deviations between predicted and actual resistance values and better capture the general behavior of the device.

These device models were then integrated into the TENNLab RAVENS neuroprocessor.^45^ RAVENS is a spiking neuromorphic processor that implements integrate-and-fire neurons with every neuron having a threshold parameter, whether or not the membrane potential will leak charge or if the neurons will have a refractory period after firing. The synapses have a weight and delay parameter with support for STDP. The device models replaced RAVENS’s STDP component to explore the impact of the proposed devices. Every time a neuron fires, the synapses that helped increase the charge within some time window (5 timesteps for this work) of the neuron fire will experience a potentiation update by applying a potentiation voltage to the device model to get a new resistance value. This value is then mapped to a value between −10 and 10 to act as the new synaptic weight for the said synapses. A depression update will also occur to the synapses that did not aid the neuron firing within the time window, thus allowing active synaptic connections to get stronger and less active connections to weaken.

Evolutionary Optimization for Neuromorphic Systems (EONS)^46^ was leveraged to train the SNNs with these device model behaviors. EONS optimizes the number of neurons, synapses, network topology, and other parameters through the principles of evolution. Depending on the application, it begins with a random population of networks with the appropriate number of input and output neurons. These networks are then evaluated and ranked based on their performance in a given task. The top performers are then chosen to act as “parents“ for the next generation of networks through a reproduction procedure. This method involves combining the parents’ genomes (which encode aspects of the networks) to produce a new child with traits for both parents. These child networks can then undergo mutations that randomly tweak aspects of their genomes to introduce more diversity into the population. This process is repeated for a given number of generations with the idea being that the final generation will outperform their predecessors. With this training methodology, we explored two types of applications: classification and control tasks. We used SciKit Learn’s Iris and Wine data sets for classification, and for the control tasks, we used OpenAI’s CartPole-v1 and BipedalWalker-v3. These are popular applications within the field that provide a strong baseline of understanding regarding the performance of the networks. Each of the four device variations (4, 10, 15, and 20 μm channel width) is trained for each application for 20 runs, each with 200 training epochs for every run. Networks without STDP (synaptic weights are static) are also trained to act as a control. The trained networks are then tested to capture the testing accuracy for classification tasks and the fitness score for the control tasks.

## Supplementary Material



## Notes

XRD data for bare LAO substrate, NNO film on LAO, and hydrogenated NNO (HNNO) on LAO; potentiation and depression curves from Figure 1d fitted with Box Lucas equations; correlation between *V*_CPD_ in KPFM data and electric field from COMSOL simulation; KPFM scans at the Au–Pd3, while the electrical programming is done at Au–Pd1; line profiles at three different locations at the junction showing the uniformity of contact potential difference (*V*_CPD_) at the channel between Au and Pd3 electrodes, which is unaffected by electrical stimulus at the Au–Pd1 junction; DFT results showing different surface terminations in the pristine NNO system; symmetric, nonpolar surfaces considered for further evaluation in the investigation of H addition of the work function of NNO; macroscopic average potential energy profile along the *z*-axis for pristine NNO slab; sinusoidal oscillations in the potential due to electron density variations within the NNO slab; work functions calculated using NNO (100) slabs that have varying concentrations of H atoms; relationship between bond ionicity and H dopant concentration in NNO; and DNN model prediction of the devices with different channel widths (PDF)

## ¶

Microsystems Division, Silicon Austria Laboratories, 9524 Villach, Austria

## References

[1] Yan, B. ; Li, Z. ; Taylor, B. ; Li, H. ; Chen, Y. Neuromorphic Computing Systems with Emerging Nonvolatile Memories: A Circuits and Systems Perspective. In 2020 International Symposium on VLSI Technology, Systems and Applications, VLSI-TSA 2020, 2020; pp 122–123.

[2] Hendy H., Merkel C. (2022). Review of Spike-Based
Neuromorphic Computing for Brain-Inspired
Vision: Biology, Algorithms, and Hardware. J.
Electron Imaging.

[3] Park J., Kumar A., Zhou Y., Oh S., Kim J.-H., Shi Y., Jain S., Hota G., Qiu E., Nagle A. L., Schuller I. K., Schuman C. D., Cauwenberghs G., Kuzum D. (2024). Multi-Level, Forming and Filament Free, Bulk Switching Trilayer RRAM
for Neuromorphic Computing at the Edge. Nat.
Commun..

[4] Sun Y., Kotiuga M., Lim D., Narayanan B., Cherukara M., Zhang Z., Dong Y., Kou R., Sun C.-J., Lu Q., Waluyo I., Hunt A., Tanaka H., Hattori A. N., Gamage S., Abate Y., Pol V. G., Zhou H., Sankaranarayanan S. K. R. S., Yildiz B., Rabe K. M., Ramanathan S. (2018). Strongly Correlated
Perovskite Lithium Ion Shuttles. Proc. Natl.
Acad. Sci. U.S.A..

[5] Kreuer K.-D. (1996). Proton
Conductivity: Materials and Applications. Chem.
Mater..

[6] Zhou X., Li H., Meng F., Mao W., Wang J., Jiang Y., Fukutani K., Wilde M., Fugetsu B., Sakata I., Chen N., Chen J. (2022). Revealing
the Role of Hydrogen in
Electron-Doping Mottronics for Strongly Correlated Vanadium Dioxide. J. Phys. Chem. Lett..

[7] Middey S., Chakhalian J., Mahadevan P., Freeland J. W., Millis A. J., Sarma D. D. (2016). Physics of Ultrathin
Films and Heterostructures of
Rare-Earth Nickelates. Annu. Rev. Mater. Res..

[8] Zhang Z., Sun Y., Zhang H. T. (2022). Quantum
Nickelate Platform for Future Multidisciplinary
Research. J. Appl. Phys..

[9] Bian Y., Li H., Yan F., Li H., Wang J., Zhang H., Jiang Y., Chen N., Chen J. (2022). Hydrogen Induced Electronic
Transition within Correlated Perovskite Nickelates with Heavy Rare-Earth
Composition. Appl. Phys. Lett..

[10] Oh C., Heo S., Jang H. M., Son J. (2016). Correlated Memory Resistor in Epitaxial
NdNiO_3_ Heterostructures with Asymmetrical Proton Concentration. Appl. Phys. Lett..

[11] Sidik U., Hattori A. N., Li H.-B., Nonaka S., Osaka A. I., Tanaka H. (2023). Strain Effect on Proton-Memristive
NdNiO_3_ Thin Film Devices. Appl. Phys.
Express.

[12] Chen H., Dong M., Hu Y., Lin T., Zhang Q., Guo E.-J., Gu L., Wu J., Lu Q. (2022). Protonation-Induced
Colossal Chemical Expansion and Property Tuning in NdNiO_3_ Revealed by Proton Concentration Gradient Thin Films. Nano Lett..

[13] Shi J., Zhou Y., Ramanathan S. (2014). Colossal Resistance Switching and
Band Gap Modulation in a Perovskite Nickelate by Electron Doping. Nat. Commun..

[14] Yuan Y., Patel R. K., Banik S., Reta T. B., Bisht R. S., Fong D. D., Sankaranarayanan S. K.
R. S., Ramanathan S. (2024). Proton Conducting
Neuromorphic Materials and Devices. Chem. Rev..

[15] Sidik U., Hattori A. N., Rakshit R., Ramanathan S., Tanaka H. (2020). Catalytic Hydrogen Doping of NdNiO_3_ Thin
Films under Electric Fields. ACS Appl. Mater.
Interfaces.

[16] Pati S. P., Yajima T. (2024). Review of Solid-State Proton Devices for Neuromorphic
Information Processing. Jpn. J. Appl. Phys..

[17] Sidik U., Hattori A. N., Hattori K., Alaydrus M., Hamada I., Pamasi L. N., Tanaka H. (2022). Tunable Proton
Diffusion in NdNiO_3_ Thin Films under Regulated Lattice
Strains. ACS Appl. Electron. Mater..

[18] Gamage S., Manna S., Zajac M., Hancock S., Wang Q., Singh S., Ghafariasl M., Yao K., Tiwald T. E., Park T. J., Landau D. P., Wen H., Sankaranarayanan S. K. R. S., Darancet P., Ramanathan S., Abate Y. (2024). Infrared Nanoimaging
of Hydrogenated Perovskite Nickelate Memristive Devices. ACS Nano.

[19] Pofelski A., Jia H., Deng S., Yu H., Park T. J., Manna S., Chan M. K. Y., Sankaranarayanan S. K.
R. S., Ramanathan S., Zhu Y. (2024). Subnanometer Scale Mapping of Hydrogen Doping in Vanadium Dioxide. Nano Lett..

[20] Zhou X., Li H., Jiao Y., Zhou G., Ji H., Jiang Y., Xu X. (2024). Hydrogen-Associated Multiple Electronic
Phase Transitions for D-Orbital
Transitional Metal Oxides: Progress, Application, and Beyond. Adv. Funct. Mater..

[21] Matsuzawa I., Ozawa T., Nishiya Y., Sidik U., Hattori A. N., Tanaka H., Fukutani K. (2023). Controlling Dual Mott States by Hydrogen
Doping to Perovskite Rare-Earth Nickelates. Phys. Rev. Mater..

[22] Kim Y. M., Lee J., Jeon D. J., Oh S. E., Yeo J. S. (2021). Advanced Atomic
Force Microscopy-Based Techniques for Nanoscale Characterization of
Switching Devices for Emerging Neuromorphic Applications. Applied Microscopy.

[23] Tsukahara D., Baba M., Honda S., Imai Y., Hara K. O., Usami N., Toko K., Werner J. H., Suemasu T. (2014). Potential
Variations around Grain Boundaries in Impurity-Doped BaSi2 Epitaxial
Films Evaluated by Kelvin Probe Force Microscopy. J. Appl. Phys..

[24] Balke N., Maksymovych P., Jesse S., Kravchenko I. I., Li Q., Kalinin S. V. (2014). Exploring
Local Electrostatic Effects with Scanning
Probe Microscopy: Implications for Piezoresponse Force Microscopy
and Triboelectricity. ACS Nano.

[25] Jakob D. S., Wang H., Xu X. G. (2020). Pulsed
Force Kelvin Probe Force Microscopy. ACS Nano.

[26] Nonnenmacher M., O’Boyle M. P., Wickramasinghe H. K. (1991). Kelvin Probe Force Microscopy. Appl. Phys. Lett..

[27] Melitz W., Shen J., Kummel A. C., Lee S. (2011). Kelvin Probe Force
Microscopy and Its Application. Surf. Sci. Rep..

[28] Meoded T., Shikler R., Fried N., Rosenwaks Y. (1999). Direct Measurement
of Minority Carriers Diffusion Length Using Kelvin Probe Force Microscopy. Appl. Phys. Lett..

[29] Berger R., Butt H. J., Retschke M. B., Weber S. A. L. (2009). Electrical Modes
in Scanning Probe Microscopy. Macromol. Rapid
Commun..

[30] Baumgart C., Helm M., Schmidt H. (2009). Quantitative Dopant Profiling in
Semiconductors: A Kelvin Probe Force Microscopy Model. Phys. Rev. B.

[31] Liu J., Jin J., Yang Z., Cai J., Yue J., Impundu J., Liu H., Wei H., Peng Z., Li Y. J., Sun L. (2020). Extremely
Low Program Current Memory Based on Self-Assembled All-Inorganic Perovskite
Single Crystals. ACS Appl. Mater. Interfaces.

[32] Wang J., Lv Z., Xing X., Li X., Wang Y., Chen M., Pang G., Qian F., Zhou Y., Han S. (2020). Optically
Modulated Threshold Switching in Core–Shell Quantum Dot Based
Memristive Device. Adv. Funct. Mater..

[33] Li D., Wu B., Zhu X., Wang J., Ryu B., Lu W. D., Lu W., Liang X. (2018). MoS _2_ Memristors
Exhibiting Variable Switching
Characteristics toward Biorealistic Synaptic Emulation. ACS Nano.

[34] Kumar M., Lim J., Park J. Y., Kim S., Seo H. (2020). Electric-Field-Induced
Healing of Inanimate Topographies: Multistate Resistive Switching
and Nano-Sized Artificial Synapse Functionality. Appl. Surf. Sci..

[35] Schumacher Z., Miyahara Y., Spielhofer A., Grutter P. (2016). Measurement of Surface
Photovoltage by Atomic Force Microscopy under Pulsed Illumination. Phys. Rev. Appl..

[36] Pam M. E., Li S., Su T., Chien Y.-C., Li Y., Ang Y. S., Ang K.-W., Pam M. E., Li S., Chien Y.-C., Li Y., Ang K.-W., Su T., Ang Y. S. (2022). Interface-Modulated
Resistive Switching in Mo-Irradiated ReS_2_ for Neuromorphic
Computing. Adv. Mater..

[37] Bisht R. S., Park J., Yu H., Wu C., Tilak N., Rangan S., Park T. J., Yuan Y., Das S., Goteti U., Yi H. T., Hijazi H., Al-Mahboob A., Sadowski J. T., Zhou H., Oh S., Andrei E. Y., Allen M. T., Kuzum D., Frano A., Dynes R. C., Ramanathan S. (2023). Spatial Interactions in Hydrogenated Perovskite Nickelate
Synaptic Networks. Nano Lett..

[38] Sun Y., Wang Q., Park T. J., Gage T. E., Zhang Z., Wang X., Zhang D., Sun X., He J., Zhou H., Lim D. G., Huang C., Yu H., Chen X., Wang H., Mei J., Deguns E., Ramanathan S. (2021). Electrochromic Properties of Perovskite NdNiO_3_ Thin Films for Smart Windows. ACS Appl. Electron.
Mater..

[39] Tilak N., Li G., Taniguchi T., Watanabe K., Andrei E. Y. (2023). Moiré Potential,
Lattice Relaxation, and Layer Polarization in Marginally Twisted MoS_2_ Bilayers. Nano Lett..

[40] Perdew J. P., Burke K., Ernzerhof M. (1996). Generalized
Gradient Approximation
Made Simple. Phys. Rev. Lett..

[41] Jacobs R., Booske J., Morgan D. (2016). Understanding and Controlling
the
Work Function of Perovskite Oxides Using Density Functional Theory. Adv. Funct. Mater..

[42] Skriver H. L., Rosengaard N. M. (1992). Surface Energy and Work Function of Elemental Metals. Phys. Rev. B.

[43] Ji D. P., Zhu Q., Wang S. Q. (2016). Detailed
First-Principles Studies on Surface Energy
and Work Function of Hexagonal Metals. Surf.
Sci..

[44] Wang J., Wang S. Q. (2014). Surface Energy and
Work Function of Fcc and Bcc Crystals:
Density Functional Study. Surf. Sci..

[45] Foshie A. Z., Plank J. S., Rose G. S., Schuman C. D. (2023). Functional Specification
of the RAVENS Neuroprocessor. arXiv.

[46] Schuman C., Patton R., Kulkarni S., Parsa M., Stahl C., Haas N. Q., Mitchell J. P., Snyder S., Nagle A., Shanafield A., Potok T. (2022). Evolutionary vs Imitation Learning
for Neuromorphic Control at the Edge. Neuromorphic
Computing and Engineering.

