# Supplementary material for: Kelvin Probe Force Microscopy Imaging of Plasticity in Hydrogenated Perovskite Nickelate Multilevel Neuromorphic Devices
Source: ACS Nano. 2025 Feb 11;19(7):6815–25. doi: 10.1021/acsnano.4c11567 (PMC12333431; doi:10.1021/acsnano.4c11567)
Supplement: Supplementary file 1 [file nn4c11567_si_001.pdf]

Supporting Information

Kelvin Probe Force Microscopy Imaging of Plasticity in Hydrogenated Perovskite Nickelate Multilevel Neuromorphic Devices

*Tamal Dey[1], Xinyuan Lai[2], Sukriti Manna[3], Karan Patel[4], Ranjan Kumar Patel[1], Ravindra Singh Bisht[1, ¥], Yue Zhou[5], Shaan Shah[5], Eva Y. Andrei[2], Subramanian K.R.S. Sankaranarayanan[3], Duygu Kuzum[5], Catherine Schuman[4] and Shriram Ramanathan[1, *]*

[1]*Department of Electrical and Computer Engineering, Rutgers University, Piscataway, New Jersey 08854, United States*

[2]*Department of Physics and Astronomy, Rutgers University, Piscataway, New Jersey 08854, United States*

[3]*Center for Nanoscale Materials, Argonne National Laboratory, Lemont, Illinois 60439, United States and Department of Mechanical and Industrial Engineering, University of Illinois, Chicago, Illinois 60607, United States*

[4]*Department of Electrical Engineering & Computer Science, University of Tennessee, Knoxville, 1520 Middle Dr, Knoxville, TN 37996, United States*

[5]*Department of Electrical and Computer Engineering, University of California, San Diego, La Jolla, California 92093, United States*

¥ *Currently at Microsystems Division, Silicon Austria Labs, 9524 Villach, Austria*

*Corresponding author (Shriram Ramanathan) email: shriram.ramanathan@rutgers.edu

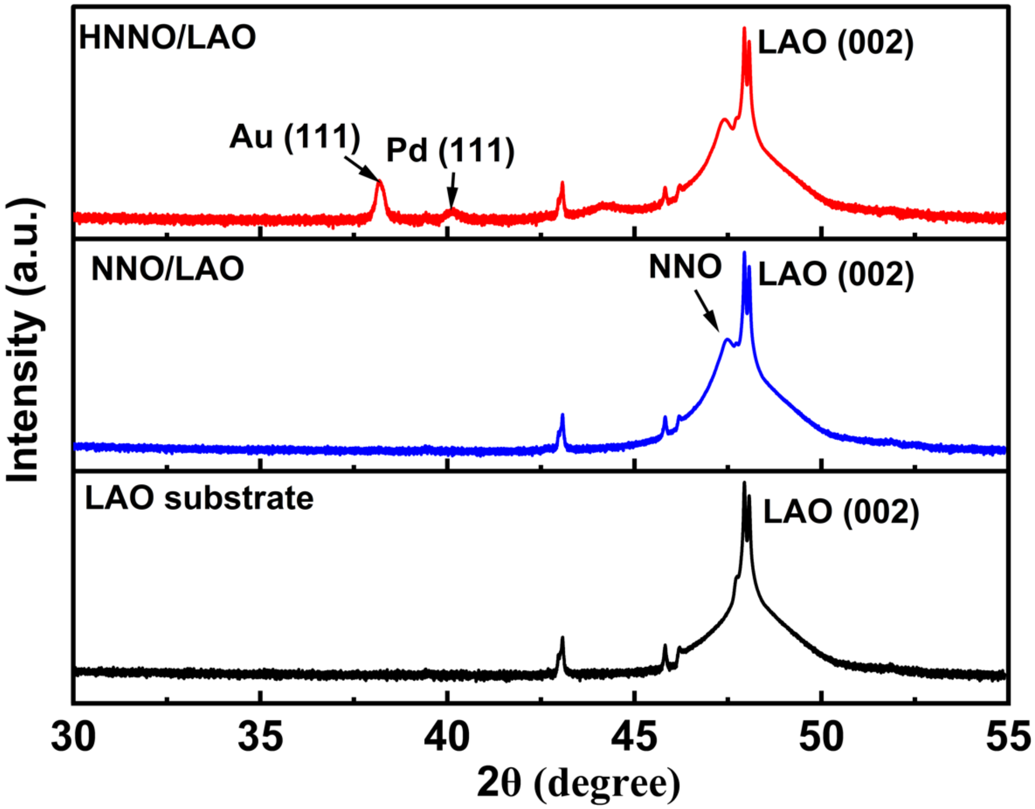

Figure S 1: X-ray diffraction data for bare LAO substrate, NNO film on LAO, and hydrogenated NNO (HNNO) on LAO. The (002) characteristic XRD peak of the LAO substrate (in black) is visible in all samples, while the characteristic NNO peak (in blue) is shifted to a slightly lower 2θ after hydrogenation (in red). Additionally, Au and Pd XRD peaks are visible in HNNO sample from the lithography patterns.

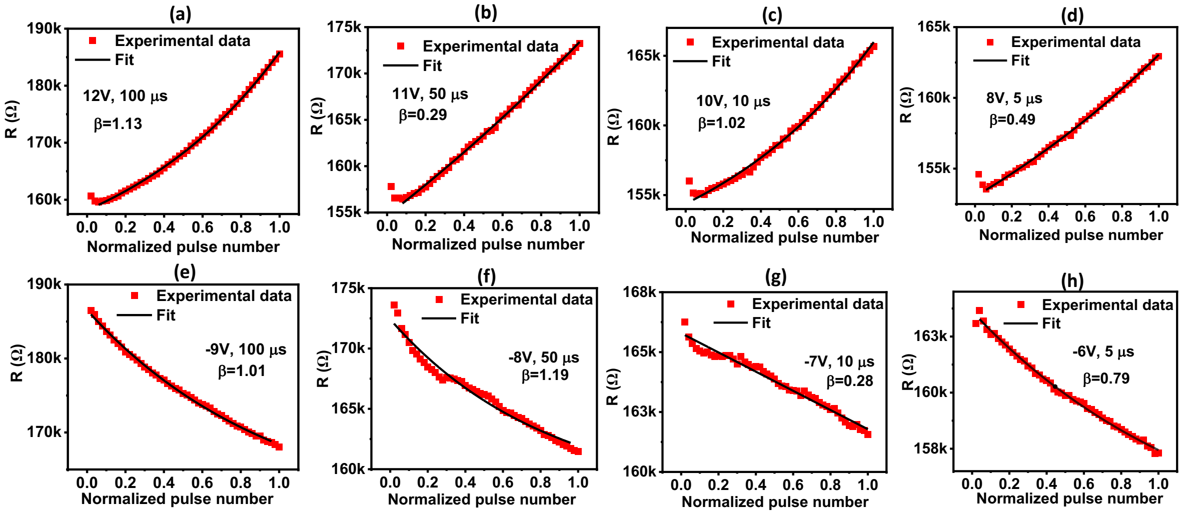

Figure S 2: (a-d) Potentiation and (e-h) depression curves from Figure 1(d) of the main manuscript fitted with Box-Lucas equations described in main manuscript equations 1 and 2. As the value of β reduces, the data trends towards linear behavior.

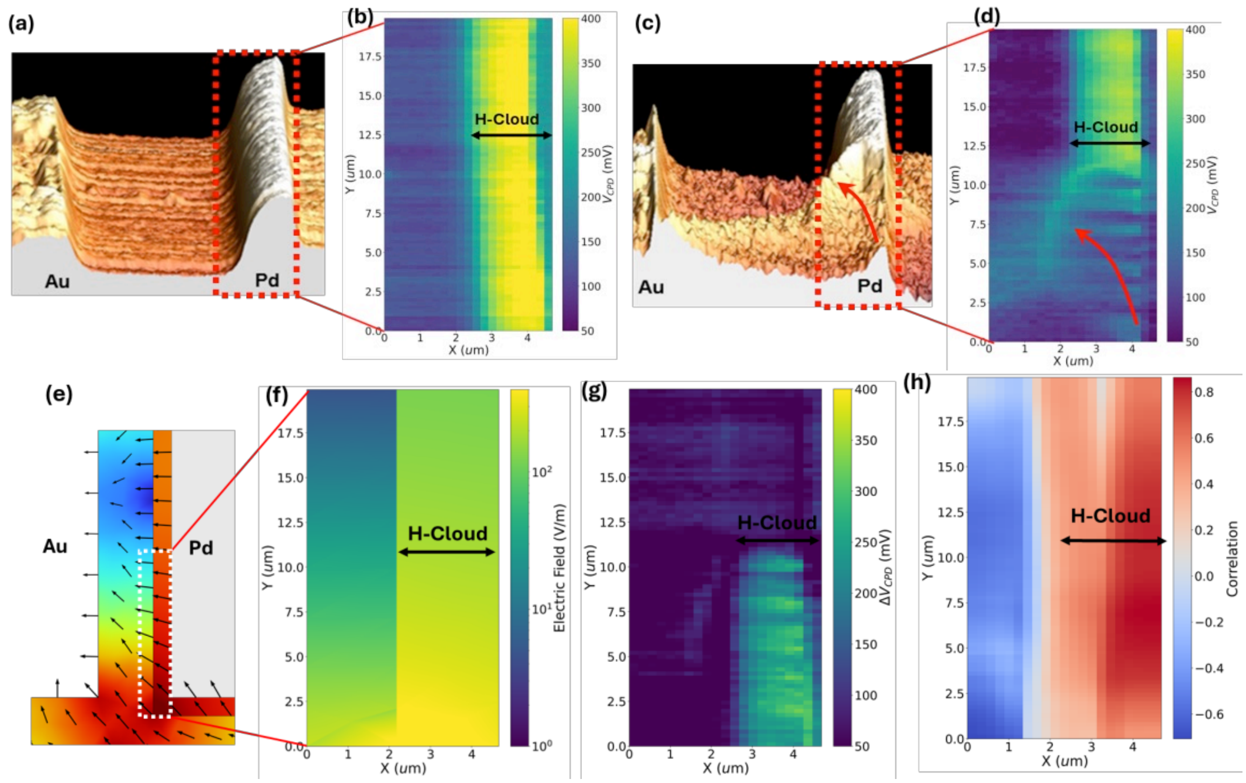

Figure S 3: KPFM scans of Au-Pd2 junction (discussed in Figure 3a and 3b in main manuscript) before and after programming, a) KPFM scan of a pristine device before programming. b) A 2D top-view map of the KPFM scan in with a magnified view of contact potential difference (V_{CPD}) in the region surrounding the Pd electrode. c) KPFM scan of the same device after programming. d) A 2D top-view map of the KPFM scan in with a magnified view of contact potential difference (V_{CPD}) in the region surrounding the Pd electrode. e) COMSOL simulation of the electric field distribution during programming. f) Zoomed-in view of the electric field distribution in (a), showing the electric field magnitude near the bottom-left corner of the Pd electrode. g) A 2D top-view map of the change in contact potential difference (ΔV_{CPD}) before and after programming. h) Statistical correlation between the electric field magnitude and the change in contact potential difference (ΔV_{CPD})

Figure S3a illustrates the KPFM scan of Au-Pd2 junction before the application of electric pulse. Figure S3b provides a 2D top-view map of the KPFM scan with a magnified view of the contact potential difference (V_{CPD}) in the region surrounding the Pd electrode, highlighted by the red dashed rectangle in Figure S3a. Figure S3c depicts the KPFM scan of the same device after application of 20 electrical pulses of 10V bias and 10 µs width, while Figure S3d provides a 2D top-view map of the same KPFM scan near the Pd electrode. As detailed in the main paper, the application of the electrical pulse train causes the proton cloud near the Pd-Au junction to shift upward and leftward; this shift is highlighted with the red arrow.

The electric field calculated using COMSOL is shown in Figure S3e. As shown, the direction of the electric field in the proton cloud at the Pd2-Au junction is upward and leftward, which aligns with the observed migration of the proton cloud in the KPFM scan after programming. To further investigate a quantitative agreement between the KPFM scans and COMSOL simulations, we zoomed into the bottom left corner of the Pd electrode. We plotted the magnitude of the electric field simulated with COMSOL (Figure S3f). We also plotted the change in contact potential difference (ΔV_{CPD}) in Figure S3g around the Pd electrode as a result of programming by calculating the difference between Figures S3b and S3d. We expect the change in contact potential difference in KPFM results to correlate with the magnitude of the electric field. Figure S3h shows the correlation between the electric field magnitude (Figure S3f) and the change in contact potential difference (Figure S3g). A strong correlation is observed between the magnitude of the applied electric field and V_{CPD} in the proton cloud, indicating good agreement between the KPFM measurements and the COMSOL simulations. The correlation is higher near the vicinity of the Pd electrode, which implies the applied electric field resulted in proton displacement in the heavily doped regions.

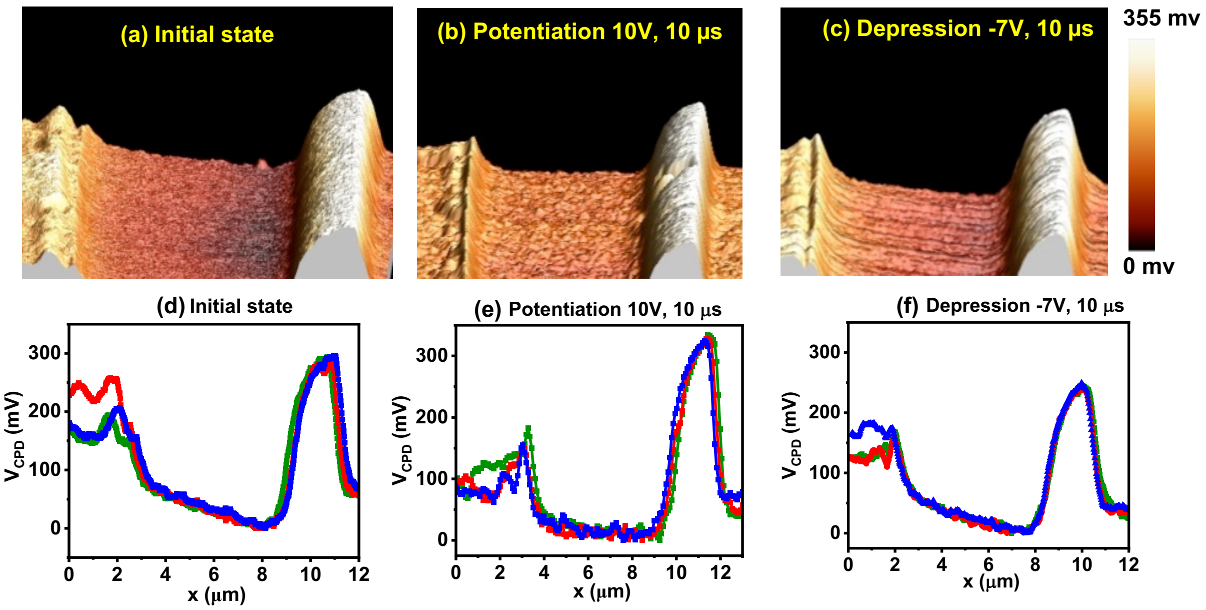

Figure S 4: KPFM scan at the Au-Pd3 junction, while the electrical programming is done at Au-Pd1. Left side and right side of the KPFM maps correspond to regions surrounding Au and Pd3 electrodes, respectively. Mapping of contact potential difference (V_{CPD}) between the KPFM tip and the sample visualized for (a) the pristine sample prior to any electrical programming, as termed as 'initial state,' (b) after applying 20 pulses of 10V height and 10 µs width, termed as 'potentiation,' (c) after applying 20 pulses of -7V height and 10 µs, termed as 'depression'. (d-f) Line profiles showing variation of V_{CPD} within the channel between Au and Pd electrodes at 3 representative positions for initial, potentiation and depression conditions each respectively.

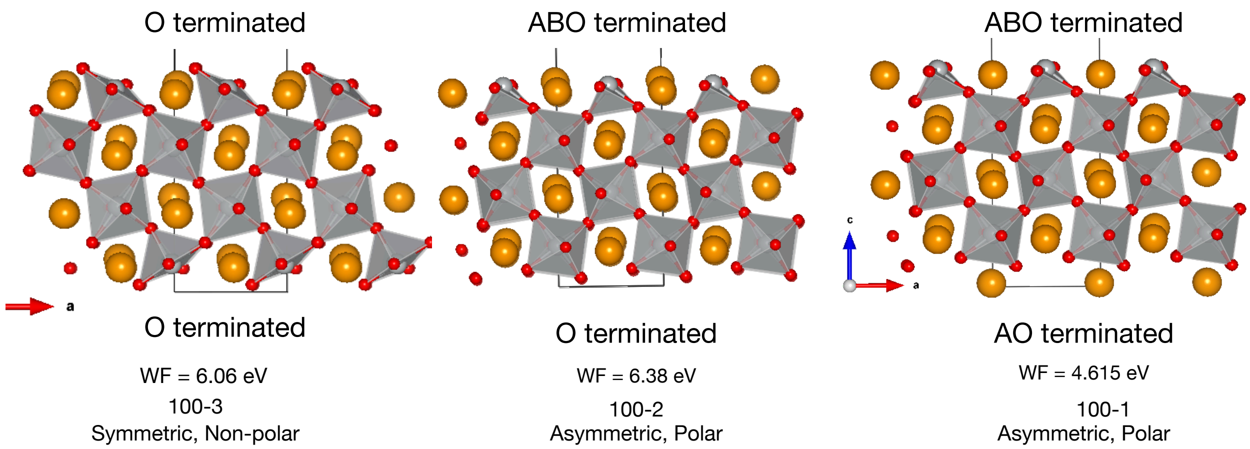

Figure S 5: Different surface terminations in pristine NNO system. Symmetric, non-polar surfaces have been considered for further evaluation in the investigation of H addition of the work function of NNO in this study.

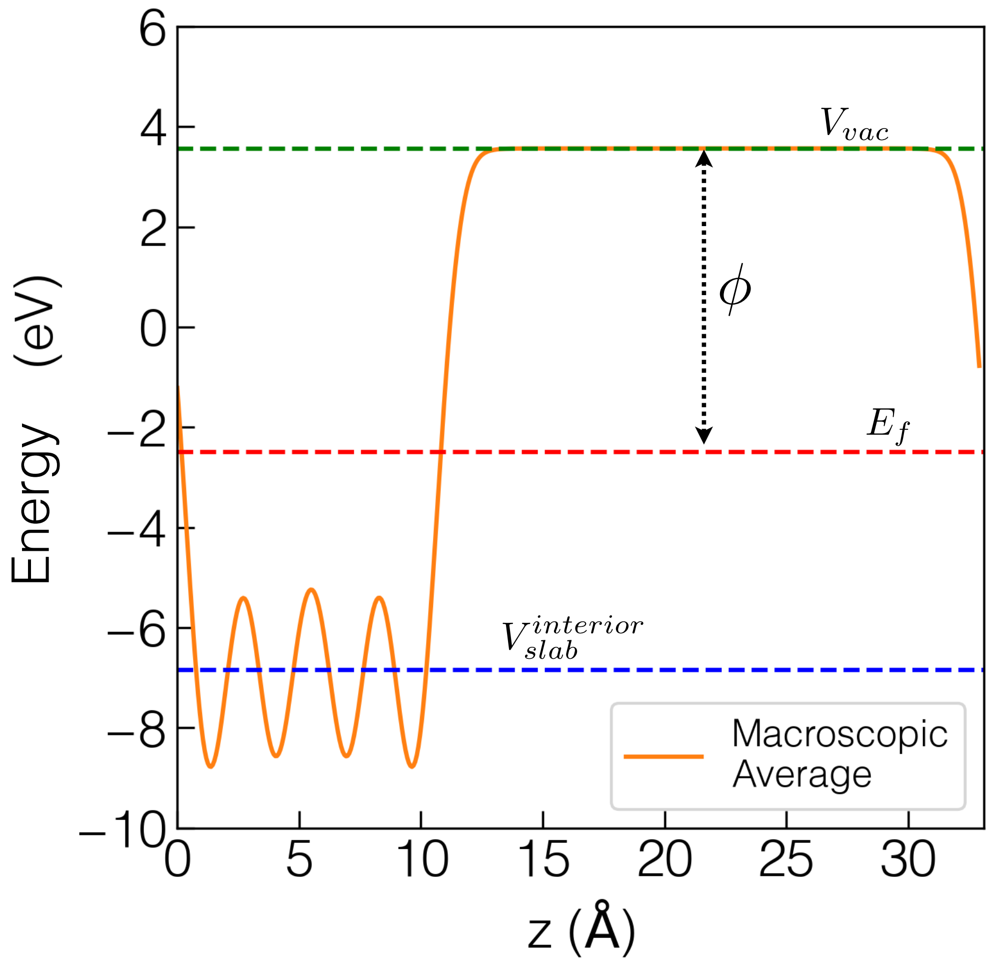

Figure S 6: Macroscopic average potential energy profile along the z-axis for pristine NNO slab. The orange curve represents the macroscopic average potential energy. The dashed red line indicates the Fermi energy (E_F), the dashed green line indicates the vacuum energy level V_{vacuum} and the dashed blue line represents the interior slab $V_{interior}$ slab. The work function (ϕ) is shown as the energy difference between the vacuum level and the Fermi level. The sinusoidal oscillations in the potential are due to electron density variations within the NNO slab.

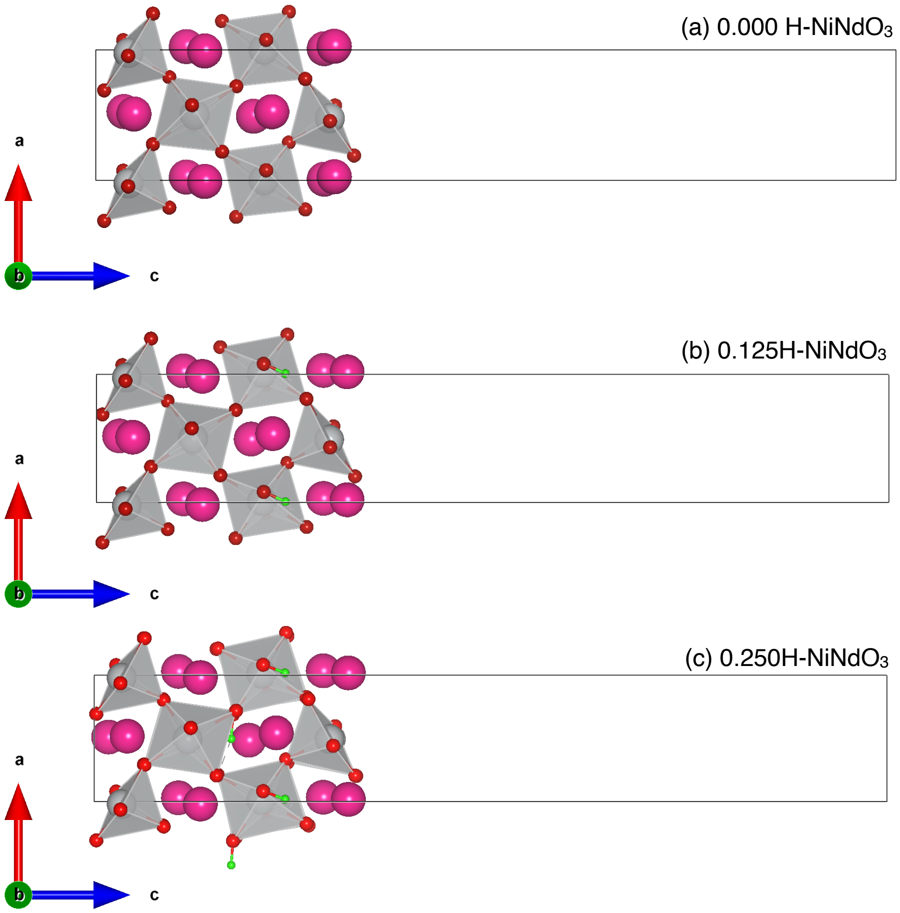

Figure S 7: The NNO (100) slab is 10 Å thick, has 8 NNO formula units, and a vacuum layer of 15 Å. Work functions are calculated using NNO (100) slabs that have varying concentrations of H atoms, including (a) the pristine, (b) 0.125 H per Ni, and (c) 0.25 per Ni.

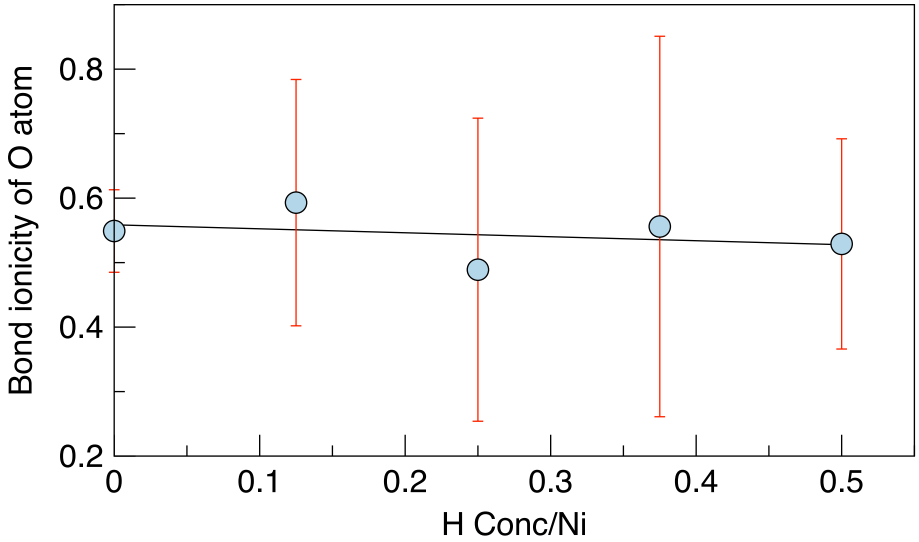

Figure S 8: Relationship between bond ionicity and H dopant concentration in NNO. The x-axis represents the H dopant concentration, while the y-axis shows the bond ionicity. Blue circles indicate the averaged values, and red vertical error bars represent the variability in Bader charges of all the oxygen atoms in the system for each concentration level. The O-bond ionicities are provided,[1] where ionicity is defined by the ratio of the computed atomic charge on oxygen to the value of -2 expected for a perfectly ionic system (e.g., an O atomic charge of -1.2 yields a bond ionicity of -1.2/-2 = 0.6, and a perfectly ionic system yields a bond ionicity of -2/-2 = 1). The atomic charges were calculated using Bader charge analysis of NNO.

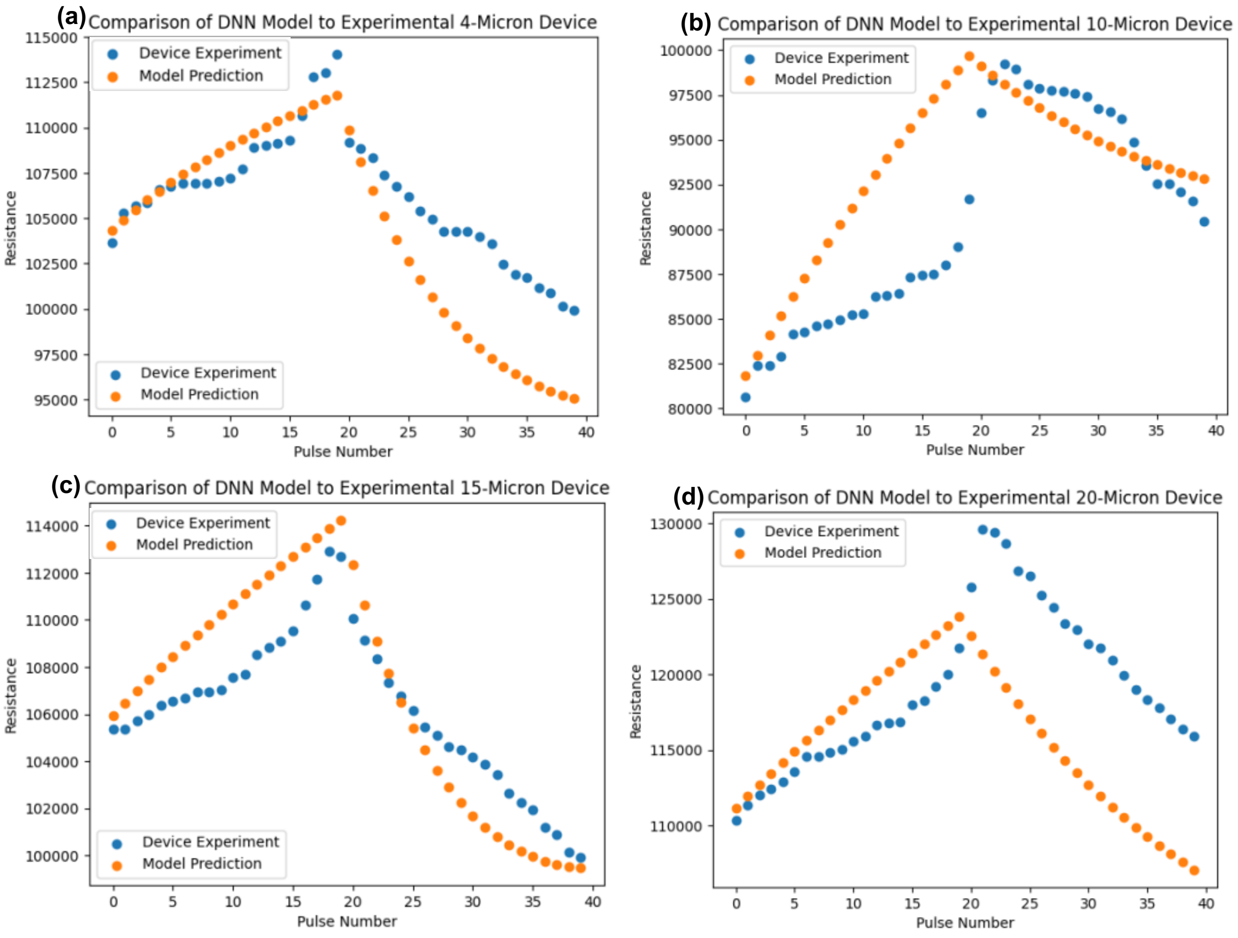

Figure S 9: Deep neural networks (DNN) model prediction of experimental data is depicted: true (in blue) and predicted data (in orange) for (a) 4 µm channel width, (b) 10 µm channel width, (c) 15 µm channel width, (b) 20 µm channel width. It may be noted that the DNN model takes previous prediction to make next prediction, explaining the deviation from experimental data.

Reference

(1) Jacobs, R.; Booske, J.; Morgan, D.; Jacobs, R.; Morgan, D.; Booske, J. Understanding and Controlling the Work Function of Perovskite Oxides Using Density Functional Theory. *Adv Funct Mater* **2016**, *26* (30), 5471–5482. https://doi.org/10.1002/ADFM.201600243.
